# Plasma Proteins at the Interface of Dental Implants Modulate Osteoblasts Focal Adhesions Expression and Cytoskeleton Organization

**DOI:** 10.3390/nano9101407

**Published:** 2019-10-02

**Authors:** Ludovica Parisi, Andrea Toffoli, Miriam Cutrera, Massimiliano G. Bianchi, Simone Lumetti, Ovidio Bussolati, Guido M. Macaluso

**Affiliations:** 1 Centro Universitario di Odontoiatria, Dipartimento di Medicina e Chirurgia, Università di Parma, 43126 Parma, Italy; andrea.toffoli@unipr.it (A.T.); miriam.cutrera@studenti.unipr.it (M.C.); simone.lumetti@unipr.it (S.L.); guidomaria.macaluso@unipr.it (G.M.M.); 2 Dipartimento di Medicina e Chirurgia, Università di Parma, 43126 Parma, Italy; massimiliano.bianchi@unipr.it (M.G.B.); ovidio.bussolati@unipr.it (O.B.); 3 Istituto dei Materiali per l’Elettronica ed il Magnetismo, Consiglio Nazionale delle Ricerche, Parco Area delle Scienze 37/A, 43124 Parma, Italy

**Keywords:** adsorption, focal adhesions, dental implant, osseointegration, osteoblasts, titanium

## Abstract

The host-material interface is a crucial relationship dictating the possibility of successful osseointegration in implant dentistry. The aim of the present study was to characterize the effects of plasma proteins pre-adsorption on the adhesion capacity of osteoblasts, which occurs immediately after implant insertion in vivo. After having pre-adsorbed human plasma proteins on a machined and microrough titanium surface, MC3T3-E1 osteoblasts adhesion was evaluated through crystal violet cell adhesion assay, immunofluorescence staining for cytoskeleton, focal adhesions and cell nuclei, and scanning electron microscopy. The pre-adsorbed protein layer markedly affected the adhesion rate of cells, as well as their morphology and the expression of focal contacts. Moreover, protein adsorption to the underlying titanium surface was found to be correlated to surface pre-wetting. Thus, the early adsorption of serum proteins to the interface of dental implants impacts cell adhesion in terms of strength and of focal adhesions expression.

## 1. Introduction

Osseointegration refers to new osteogenesis at the implant surface, which occurs after implant positioning and in the long-term, allows the direct and intimate relationship between living bone and implant material without the interposition of fibrous tissue [1]. Numerous factors may positively or negatively influence osseointegration. For example, implant design, surface treatment, wettability, and chemistry may be modulated in order to promote osseointegration, with numerous evidence from the literature supporting the use of microrough surfaces over smooth counterparts, and of hydrophilic vs. hydrophobic [2,3,4]. Nevertheless, the underlying molecular mechanisms of osseointegration are still largely unexplored.

In particular, a scarcely studied aspect of osseointegration involves the adsorption of plasma proteins on the implant surface, which occurs shortly after insertion as a consequence of the implant soaking with a patient’s blood [5]. As plasma contains a vast array of proteins with different structures and functions, such as inflammatory cytokines, chemokines, growth factors, and matrix components, protein adsorption provides a plethora of molecular cues to the colonizing cells. As such, it is reasonable to consider protein surface conditioning as of pivotal importance for osseointegration [6].

The new osteogenesis at the implant surface requires the adhesion of osteoprogenitors. Therefore, the factors influencing cell adhesion are crucial, including accurate information regarding the role played by adsorbed proteins. The differences in osteoblast responses on the various types of titanium surfaces that have been proposed for dental implants might be related to this aspect. Furthermore, it has been demonstrated that specific serum proteins, i.e., fibronectin and vitronectin, are essential for the attachment and spreading of bone-forming cells, and that depletion of specific serum proteins may greatly inhibit cell attachment and spreading on biomaterial surfaces [7,8,9,10].

Considering these premises, the aim of the present study has been to accurately investigate how plasma proteins adsorbed at the interface of machined and microrough titanium modulate the early stages of osteoblast response, including adhesion, expression of focal contacts, cytoskeleton organization, and the interaction of cells with the underlying surface.

## 2. Materials and Methods

To study the affinity of machined and microrough titanium surfaces for plasma proteins, a Bradford assay has been developed. Secondly, since new osteogenesis by implant interface first requires the adhesion of osteoprogenitors an adhesion assay, a fluorescence staining for cell cytoskeleton, focal adhesions and nuclei, and a qualitative scanning electron microscopy coupled to focused ion beam (FIB) analysis have been conducted.

### 2.1. Titanium Discs

Commercially pure, grade 4 (ISO5832/2) titanium discs (diameter 8.0 mm; thickness 3.5 mm) were used (Sweden&Martina SpA, Due Carrare, PD, Italy). Discs presented either a machined or microrough surface profile, which was obtained through acid-etching and sandblasting (ZirTi^®^, Sweden&Martina). All the titanium discs were cleaned in an Argon-activated plasma reactor and sterilized by beta rays, following the same procedure used for commercial implants. According to the manufacturer’s information, beta rays have been chosen as a method for sterilization because the process is rapid, reliable, and does not require the presence of radioactive sources, thus avoiding the formation of toxic compounds. The sterilization procedures have been carried out in accordance with UNI EN ISO 13,485 and UNI EN ISO 9001.

### 2.2. Protein Adsorption

#### 2.2.1. Surface Pre-Conditioning

To allow the formation of a plasma protein microfilm layer at the interface, titanium discs were soaked in a 300 µg/mL fetal bovine serum solution (FBS, Thermo Fisher Scientific, Waltham, MA, USA) prepared in phosphate-buffered saline (PBS, Thermo Fisher Scientific, Waltham, MA, USA) at 37 °C and 5% CO_2_ for 1 h. At the end of the incubation time, softly bound proteins were removed with two rinses in PBS.

#### 2.2.2. Bradford Assay

Bradford assay was used to quantitatively investigate the amount of proteins adsorbed on the different titanium surfaces. In brief, after surface pre-conditioning, the amount of non-adsorbed proteins was quantified through the Bradford assay (BIO-RAD Protein Assay, BIO-RAD, Hercules, CA, USA) following the manufacturer’s recommendation. Ten microliters of supernatants (including PBS washings) were mixed with 200 µl of Bradford solution and after a 2 min incubation at 37 °C, sample absorbance was measured at 620 nm with a Multiskan FC plate reader (Thermo Fisher Scientific, Waltham, MA, USA). The amount of adsorbed proteins was finally calculated by subtracting protein residual concentration in the final supernatant and in the washings from the initial one.

### 2.3. Osteoblasts Adhesion Analysis

#### 2.3.1. Cell Culture

Murine MC3T3-E1 osteoblastic cells were obtained from the American Type Culture Collection (CRL-2593). Cells were cultured in complete alpha-MEM (Alpha-MEM, Thermo Fisher Scientific, Waltham, MA, USA) supplemented with 10% FBS and 1% penicillin and streptomycin (PenStrep, Thermo Fisher Scientific, Waltham, MA, USA).

Upon confluence, cells were trypsinized and seeded at a density of 10,000 cells/disc on titanium discs positioned in 48-well plates (JET BIOFIL, Guangzhou, China) and pre-conditioned as reported in Section 2.3.1.

To avoid the possibility that the presence of 10% FBS in the culturing medium masked the effects of surface conditioning during the experiments, cells were cultured in complete alpha-MEM additioned with 0.5% FBS and 1% PenStrep.

#### 2.3.2. Surface Conditioning

Ten% FBS in PBS or plain PBS (control) were used to condition titanium surfaces before cell seeding. In brief, titanium discs were soaked for 1 h at 37 °C and 5% CO_2_ and then washed twice in PBS, as it has been reported in Section 2.2.1.

#### 2.3.3. Adhesion Assay

The adhesion rate of MC3T3-E1 cells, cultured on titanium discs pre-conditioned with serum proteins, was determined by staining adherent cells with crystal violet (CV, Sigma-Aldrich, Saint Louis, MI, USA).

Cells were plated on titanium discs and their adhesion was evaluated 1, 3, and 6 h after seeding. In brief, after the removal of culture medium, cells were washed twice in PBS, fixed in 4% paraformaldhyde (PFA, Sigma-Aldrich, Saint Louis, MI, USA) for 20 min at RT and stained with 0.1% CV solution for 30 min at RT. After extensive washing in deionized H_2_O to remove CV excess, the dye bound to adherent cells was solubilized with a 10% acetic acid solution (Sigma-Aldrich, Saint Louis, MI, USA) for 5 min at RT, and the absorbance of eluate was measured at 620 nm using a Multiskan FC plate reader (Thermo Fisher Scientific, Waltham, MA, USA).

#### 2.3.4. Cell Morphology and Focal Adhesion Expression

Cell morphology and focal adhesion expression were investigated after 24 h of culture by immunofluorescence.

Cells were fixed in a 4% PFA solution (Sigma-Aldrich, Saint Louis, MI, USA) for 10 min at RT, then rinsed twice in PBS and permeabilized with a 0.1% *v/v* Triton-X solution (Triton-X, Sigma-Aldrich, Saint Louis, MI, USA) for 5 min at RT. To block non-specific sites, samples were washed twice in PBS and incubated in a 1% bovine serum albimin solution (BSA, Sigma-Aldrich, Saint Louis, MI, USA) for 30 min at RT. Focal adhesions were labeled with an anti-vinculin monoclonal antibody, clone 7F9 (FAK100, Merck Millipore, Darmstadt, Germany) at a dilution of 1:100 in 1% BSA for 1 h at RT. Antibody positivity was subsequently revealed with a secondary AlexaFluor^®^488 anti-mouse antibody at a dilution of 1:200 in PBS. TRITC-conjugated phalloidin (FAK100, Merck Millipore, Darmstadt, Germany, 1:200) was simultaneously used to stain actin cytoskeleton. Eventually, after 3 rinses in PBS, cell nuclei were stained with DAPI 1:1000 in PBS (FAK100, Merck Millipore, Darmstadt, Germany) for 5 min at RT.

Images were taken with a stereomicroscope equipped for fluorescence (SMZ25, Nikon, Tokjo, Japan) and analyzed with the NIS-Element Br5.11 Software (Nikon, Tokjo, Japan). Cell mean radius and the number of expressed focal adhesions were evaluated on 6 regions of interest (ROIs).

#### 2.3.5. Morphological Analysis of Osteoblast/Titanium Relationship

The interaction between cells and titanium surface was qualitatively investigated by scanning electron microscopy (SEM) 24 h after seeding.

Cells were prepared for the analysis as previously described [11] and analyzed using a dual-beam Zeiss Auriga Compact system equipped with a GEMINI Field-Effect SEM column and a gallium FIB source (Zeiss, Oberkochen, Germany). SEM analysis was performed at 5 keV, while the gallium ion beam for cross-sectional analysis was accelerated at 30 kV.

### 2.4. Statistical Analysis

Data were analyzed by Prism 7 (GraphPad, La Jolla, CA, USA). All the values have been reported as the means ± SD of three independent experiments performed in multiple replicates. Differences between the groups were evaluated with either *t*-test or 2-way ANOVA with Tukey’s multiple comparison *post hoc* analysis. Differences were considered significant when *p* < 0.05.

## 3. Results

### 3.1. Serum Proteins Firmly Bind to Titanium Surfaces

**Figure 1:**
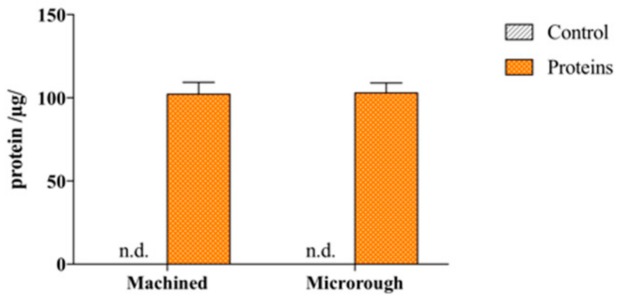
Serum protein adsorption. Amount of firmly adsorbed serum proteins to machined or microrough titanium surfaces 1 h after incubation at 37 °C and 5% ppCO_2._

The result of a Bradford assay on machined and microrough titanium after serum conditioning is shown in Figure 1.

The titanium surface with a machined profile firmly adsorbed 102.13 ± 7.23 µg of proteins (68.1% of total serum proteins), while the titanium surface with a microrough topography adsorbed 102.94 ± 6.02 µg (68.6%), showing no significant differences between the two groups (*p* = 0.9736). Interestingly, after extensive washing in PBS, only a minimal amount of proteins was detected to be released in the supernatants for both groups, 1.63 ± 0.21 µg and 1.62 ± 0.15 µg for machined and microrough topography, respectively. These data indicate a remarkable affinity of titanium for serum proteins.

### 3.2. Serum Proteins at Titanium Interface Increase the Strength of Cell Adhesion and Focal Adhesions Expression

**Figure 2:**
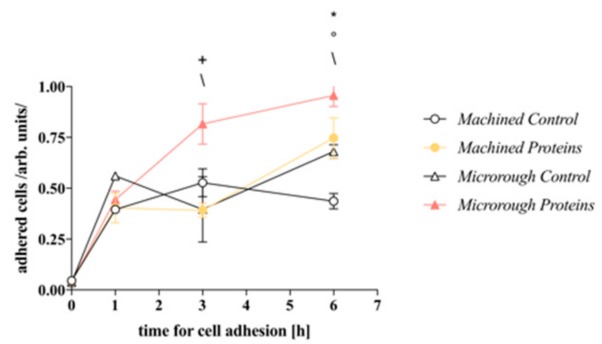
Cell adhesion. Time course of MC3T3-E1 cells adhesion to machined and microrough titanium surfaces pre-conditioned or not with serum proteins 1, 3, and 6 h after seeding; 2-way ANOVA: Machined Control vs. Machined Proteins * = *p* < 0.05; Machined Control vs. Microrough Control ° = *p* < 0.05; Machined Proteins vs. Microrough Proteins + = p<0.05; Microrough Control vs. Microrough Proteins \ = *p* < 0.05.

The influence of pre-adsorbed serum proteins on the rate of osteoblasts adhesion to the machined and microrough titanium surface is shown in Figure 2. One hour after seeding, no significant difference was detected among the groups. However, after 3 h, the presence of serum proteins pre-adsorbed on the microrough surface had already increased cell adhesion compared with the control surface (*p* = 0.0002). Furthermore, cell adhesion on the microrough surface pre-adsorbed with serum proteins was higher than cell adhesion on the serum adsorbed machined counterpart (*p* < 0.0001), indicating change in cell adhesion directly correlated to the surface microtopography. After 6 h, the difference between the microrough groups was still evident (Microrough Control vs. Microrough Proteins *p* = 0.0186) and became significant also between the machined surfaces (Machined Control vs. Machined Proteins *p* = 0.0006). Interestingly, after 6 h, a significant difference was also detected between the two control (not serum-adsorbed) surfaces (Machined Control vs. Microrough Control *p* = 0.0241), confirming the role of the surface profile in controlling osteoblasts-titanium interaction.

**Figure 3:**
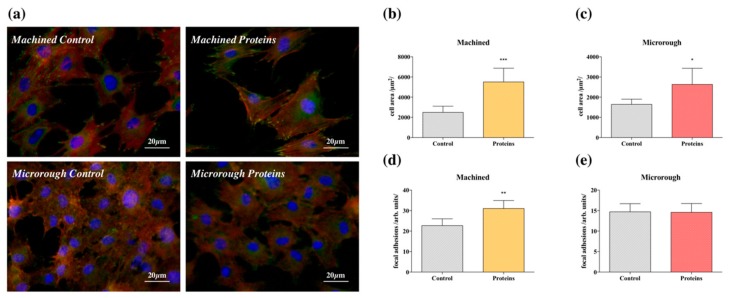
Fluorescence microscopy: (**a**) Representative immunofluorescence images showing MC3T3-E1 cells on machined and microrough titanium surfaces pre-conditioned or not with serum proteins, stained for actin (red), vinculin (green), and cell nuclei (blue) 24 h after seeding; (**b**) analysis of cell area of MC3T3-E1 cultures seeded on machined titanium surfaces. *t*-test: *** = *p* < 0.001; (**c**) analysis of cell area for MC3T3-E1 cultures seeded on microrough titanium surfaces. *t*-test: * = *p* < 0.05; (**d**) analysis of focal adhesions distribution along MC3T3-E1 cells seeded on machined titanium surfaces. *t*-test: ** = *p* < 0.01; (**e**) analysis of focal adhesions distribution along MC3T3-E1 cells seeded on microrough titanium surfaces. *t*-test: *p* > 0.05.

Immunostaining for the detection of focal adhesions was performed 24 h after seeding to further investigate the mechanisms involved in serum protein adsorption and surface topography. Representative fluorescence images are reported in Figure 3a. The cells that were grown on surfaces pre-adsorbed with proteins appeared healthy and with a quadrangular shape typical of mature osteoblasts. In contrast, cells grown on nonconditioned surfaces had a more contracted shape. This observation was validated both for the machined and the microrough surfaces and it was confirmed by cell area analysis, which is reported in Figure 3b,c (Machined Control vs. Machined Proteins *p* = 0.0006; Microrough Control vs. Microrough Proteins *p* = 0.0166). Moreover, enhanced organization of actin stress fibers was present in cells grown on the pre-treated machined surface when compared with the control counterpart. Actin filaments were mainly structured as ventral stress fibers, linking two focal adhesions at their opposite sides, which therefore resulted to be more numerous on the protein-conditioned machined surface (Figure 3d–*p* = 0.0018). A comparable difference was not detected for microrough surfaces. However, the microrough surface induced a slight blurring of the images, rendering difficult a reliable count of the focal adhesions for this surface.

### 3.3. Serum Proteins at Titanium Interface Did Not Affect Cell Morphology at SEM

Effective adhesion also requires a close interaction among the cells and the underlying titanium surface. To properly evaluate this interaction an SEM analysis has been performed.

**Figure 4:**
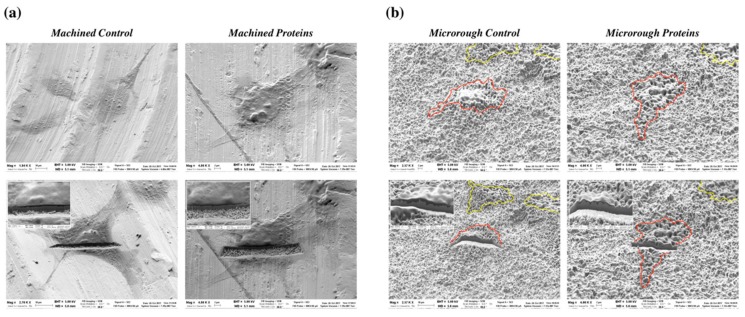
Scanning electron microscopy: (**a**) Representative SEM images showing MC3T3-E1 cells 24 h after seeding on machined titanium surfaces pre-conditioned or not with serum proteins, before and after focused ion beam (FIB) cross-sectional analysis; (**b**) representative SEM images showing MC3T3-E1 cells 24 h after seeding on microrough titanium surfaces pre-conditioned or not with serum proteins, before and after FIB cross-sectional analysis. Red and yellow lines highlight the borders of osteoblasts on titanium surfaces.

Representative SEM images are reported in Figure 4. The morphology of the adhesion of the cells to the machined surface pre-conditioned or not with serum proteins was similar, with cells displaying a flat and well-spread shape (Figure 4a). However, it is not possible to appreciate the intimate relationship that occurs between cells and the underlying surface, due to the rather smooth topography. On microrough titanium, cells also showed a comparable interaction among test and control samples, and the intimate relationship with the underlying surface was more evident (Figure 4b). Osteoblasts adhered closely to the surface and the underlying titanium micro-texture is evident under the cell soma. The FIB cross-sectional analysis confirmed SEM observations allowing a detailed visualization of how cells contact the underlying surface. The cellular morphological aspect was not related to the serum proteins pre-conditioning step but clearly influenced by the type of surface.

## 4. Discussion

Immediately after their insertion into the host’s anatomical site, implantable materials come in contact with blood; as a consequence, plasma proteins have been shown to get promptly adsorbed on biomaterial surfaces, according to their chemical-physical properties and to those of the biomaterial itself. In turn, protein adsorption has been proven to play a pivotal role in mediating cell responses to biomaterials, as adsorbed proteins can be recognized by colonizing cells, to which they can convey different biological stimuli [6]. With particular regard to endosseous dental implants, a deeper understanding of the role played by serum proteins adsorbed at the interface of titanium may provide useful insights on how to improve their biological performance.

The aim of the present study has been to investigate the role of adsorbed proteins in orchestrating osteoblasts adhesion to machined and microrough titanium. We found that two commercial titanium implant surfaces possess a great capacity to adsorb serum proteins. Furthermore, the adsorbed protein layer played a pivotal role in modulating osteoblasts adhesion to the surface, showing to be relevant for the tight adhesion of osteoblasts, with a consequent abundant expression of focal contacts. Conversely, the intimate adhesion that occurred among osteoblasts and the underlying titanium surfaces was not directly correlated to the presence of adsorbed proteins, but rather to the pre-wetting of the surface.

Regarding protein adsorption, according to some authors, titanium foil possesses a weak capacity to firmly bind serum proteins (around 20% of the total amount) [12], which can be improved by increasing the surface available for protein adsorption, i.e., developing micro- and nanostructures [12], or by changing the chemical state of the titanium dioxide layer, for example, by its thickening [13]. In this study, we have observed that both the commercially available surfaces of titanium dental implants adsorb up to 60% of the total amount of proteins (Figure 1). Consistently, when serum proteins were pre-adsorbed, the presence of an adsorbed protein layer promoted cell adhesion both on the machined and on the microrough surface (Figure 2). However, the contribution of titanium surface micro-topography in promoting the adhesion should be recognized. Even if the pre-adsorbed protein layer improved the adhesion of cells on the machined surface when compared to control, no statistically significant differences were detected among the pre-conditioned machined surface and the control microrough surface 6 h after seeding. It is reasonable to state that the texture of the microrough surface provides more anchorage points to cells [11]. This appears consistent with the wider spreading of osteoblasts and the higher expression of focal adhesions on the machined surface (Figure 3). Interestingly, even if the microrough topography blurred the focus of the image, making unreliable subsequent quantitative image analysis, the almost flat aspect of the machined surface allowed to better appreciate how proteins influenced the shape of the cells. Cells on the pre-conditioned machined surface showed evidence of well-defined cytoskeleton structures, i.e., transversal arc organization. This finding, according to the literature, is associated with the presence of plasma fibronectin on the surface [14,15]. In addition, the adsorption of fibronectin in the literature is recognized to be associated with the expression of vinculin in focal adhesions [16]. We can thus state that the adsorption of plasma proteins is important to promote strong cell adhesion, and further we can speculate the tested titanium dental implant surfaces may possess the capacity to adsorb specific serum proteins, which are essential for the attachment and spreading of bone-derived cells [10]. However, we did not investigate the composition of the protein layer adsorbed at the interface of titanium, and this is a major limitation of the present study. Moreover, even if previous papers could not detect significant differences in the chemistry of the machined and the microrough surface [17], our data cannot exclude that a particular surface profile could result in the differential adsorption of specific proteins from blood plasma. The characterization of the proteins adsorbed at the interface of the different titanium implant surfaces will surely be addressed in future studies.

Using SEM imaging coupled to FIB cross-sectional analysis, we had further qualitatively analyzed how the protein layer adsorbed at the interface influences the distance of adhering osteoblasts to the underlying titanium surface, which is considered a factor of pivotal importance to ensure proper biological stability during implant osseointegration (Figure 4). Differently from our previous published study [11], we observed that cells closely adhered to the underlying titanium surface both in the presence or in the absence of pre-adsorbed plasma proteins. In particular, in the presence of a microrough surface, cells did not grasp on titanium ridges and bridged over the substrate valleys, likely as a consequence of microbubbles entrapment [11].Therefore, quite surprisingly, our data showed that when the surface is pre-wet both in the presence or in the absence of plasma proteins, osteoblasts adhered closely to titanium, leading to glimpse its texture underneath the cell soma.

## 5. Conclusions

This study showed that plasma proteins adsorbed to both machined and microrough titanium implant interface ameliorate osteoblast adhesion.

A close and firm relationship between osteoblasts and the underlying titanium surface is a prerequisite for effective biological stability. Therefore, given that plasma proteins promote the expression of structures involved in the stable adhesion of cells to the underlying surfaces, the data presented in this study may provide the clinician further rationale for the importance to soak titanium dental implants with the patient’s own blood prior to its positioning in the alveolar process.

Taken together, our findings provide new insights into the molecular mechanisms of osseointegration.
